# Application of leaf size and leafing intensity scaling across subtropical trees

**DOI:** 10.1002/ece3.6943

**Published:** 2020-11-12

**Authors:** Jun Sun, Xiaoping Chen, Mantang Wang, Jinlong Li, Quanlin Zhong, Dongliang Cheng

**Affiliations:** ^1^ Fujian Provincial Key Laboratory of Plant Ecophysiology Fujian Normal University Fuzhou China; ^2^ School of City and Civil Engineering Zaozhuang University Zaozhuang China; ^3^ Institute of Geography Fujian Normal University Fuzhou China

**Keywords:** broad scope, functional groups, leaf size, leafing intensity, stem size

## Abstract

Understanding the scaling between leaf size and leafing intensity (leaf number per stem size) is crucial for comprehending theories about the leaf costs and benefits in the leaf size–twig size spectrum. However, the scaling scope of leaf size versus leafing intensity changes along the twig leaf size variation in different leaf habit species remains elusive. Here, we hypothesize that the numerical value of scaling exponent for leaf mass versus leafing intensity in twig is governed by the minimum leaf mass versus maximum leaf mass (*M*_min_ versus *M*_max_) and constrained to be ≤−1.0. We tested this hypothesis by analyzing the twigs of 123 species datasets compiled in the subtropical mountain forest. The standardized major axis regression (SMA) analyses showed the *M*_min_ scaled as the 1.19 power of *M*_max_ and the ‐α (−1.19) were not statistically different from the exponents of *M*_min_ versus leafing intensity in whole data. Across leaf habit groups, the *M*_max_ scaled negatively and isometrically with respect to leafing intensity. The pooled data's scaling exponents ranged from −1.14 to −0.96 for *M*_min_ and *M*_max_ versus the leafing intensity based on stem volume (LIV). In the case of *M*_min_ and *M*_max_ versus the leafing intensity based on stem mass (LIM), the scaling exponents ranged from −1.24 to −1.04. Our hypothesis successfully predicts that the scaling relationship between leaf mass and leafing intensity is constrained to be ≤−1.0. More importantly, the lower limit to scaling of leaf mass and leafing intensity may be closely correlated with *M*_min_ versus *M*_max_. Besides, constrained by the maximum leaf mass expansion, the broad scope range between leaf size and number may be insensitive to leaf habit groups in subtropical mountain forest.

## Graphical abstract

Our hypothesis successfully predicts that the scaling relationship between leaf mass and leafing intensity is constrained to be ≤−1.0. The broad scope range between leaf size and number may not be sensitive to leaf habit groups in the subtropical mountain forest.

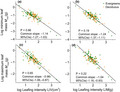

## 1 INTRODUCTION

As the terminal part of the tree canopy, current‐year shoots (i.e., twigs) sustain the static and dynamic biomechanical forces generated by leaves, flowers, and fruits (Niklas, 1992; Suzuki, 2003). At a very basic level, leaves and stems are both structurally and physiologically organographic units integrated into the context of biomass allocation (Niklas & Enquist, 2002; Sun et al., 2006; Xiang & Liu, 2009), seed and fruit size and number (Chen et al., 2009; Dombroskie et al., 2016; Leishman, 2001), and leaf size and number (Kleiman & Aarssen, 2007; Scott & Aarssen, 2012).

The trade‐off between twig leaf size and number is critical to understand as a carbon gain strategy for coexisting species. The leafing intensity premium hypothesis (LIPH, the number of leaves produced per stem tissue volume) proposed by Kleiman and Aarssen (2007) predicts the trade‐off based on a common phenomenon, that is, species with smaller leaves have more of them per stem tissue volume. According to the LIPH, the trade‐off between leaf number and stem volume emerges from the fact that small but numerous leaves provide a selective advantage over the course of plant evolution, that is, maintaining a high number of axillary buds per shoot. Previous studies have shown that the negative scaling relationship between leaf size and leafing intensity widely exists in different habitats (i.e., spatial scale) (Fajardo, 2016; Xiang, Wu, & Sun, 2010; Yang, Li, & Sun, 2008), different forest successional series (Yan, Wang, Chang, & He, 2013), and different canopy light environments (Dombroskie & Aarssen, 2012; see also, Huang et al., 2016; Fan et al., 2017). Interestingly, this negative isometric relationship is not always constant but seems to be of broad scope. For example, Milla (2009) reports the scaling exponents of mean leaf mass versus leafing intensity is lower in evergreens (*α* = −1.19) than deciduous (*α* = −1.01). And this disruption might be attributed to the integration of foliage functioning does not occur at the current‐year shoot level in evergreen (Milla, 2009; Sprugel et al., 1991). Similarly, Yang et al. (2008) report the evergreen y‐intercept of leaf area versus leafing intensity is lowest among the evergreen, deciduous, and compound‐leaved species groups. They suggest that the differences in the y‐intercepts of such scaling might account for differences in leaf mass per leaf area (LMA) between functional groups. However, given that the LMA is higher in evergreen than deciduous (Li et al., 2013) the latter usually invest more resources in the growth of stem than leaves (Li et al., 2008). This opposite trade‐off might weaken the divergence for the relationship between leaf size and number between evergreen and deciduous. Thus, we need an alternative way to quantify the broad scaling scope between leaf size and leafing intensity in different leaf habit species group.

Besides, the methods of leafing intensity and leaf size calculations are still controversial. The leafing intensity is commonly calculated as the number of leaves per unit stem volume or mass (e.g., LIV and LIM, respectively). However, this can result in a statistical bias because mean leaf mass is calculated as the total leaf mass divided by the total leaf number, whereas leafing intensity is calculated as the leaf number per unit stem volume or mass (Fajardo, 2016; Kleiman & Aarssen, 2007; Xiang et al., 2010; Yang et al., 2008). Another method uses the mean leaf mass as leaf size, while it does not truly reflect the scaling relationships between leaf size variation and leafing intensity in twig. Because maximum leaf mass or minimum leaf mass in twigs may reflect a localized departure from the normal growth rate with respect to mean leaf mass, the scaling scope of leaf size–leafing intensity may correlate with the leaf mass variations, affecting the potential carbon gain and axillary buds compensation mechanism in trees.

On the one hand, a twig has either many small leaves or a few big leaves per stem. It is well known that the minimum leaf mass might indicate the minimum carbon gain requirements of a plant. For instance, the positive net assimilation of atmospheric CO_2_ in twigs is restricted by the smallest leaf mass (Comstock & Ehleringer, 1990). Meanwhile, the small leaves usually expand in a shorter time than large leaves to reduce herbivore damage (Moles & Westoby, 2000). Thus, most woody species may have small leaves because the high leafing intensity is usually more adaptive than lower leafing intensity (Kleiman & Aarssen, 2007). On the other hand, given that the leaf supports investments (Milla & Reich, 2007; Poorter & Rozendaal, 2008), leafing intensity may decrease with increasing leaf mass (Xiang & Liu, 2009). Therefore, the trade‐off between minimum leaf mass and maximum leaf mass in twig may help understand why the leafing intensity seems to be of broad scope.

Furthermore, the leaf–stem growth hypothesis showed that the scaling for the trade‐off between individual leaf mass and leafing intensity is governed by –*α*, where *α* is the exponents of total leaf mass versus stem mass (Sun et al., 2019a). Therefore, the isometric trade‐off in leaf size/number may deduce from the tightly isometric relationship between leaf and stem (Yang et al., 2008). Mathematically, the maximum leaf mass in twig can have a statistically significant effect on the total leaf mass (*M*_leaf_) and thus leafing intensity. Recent studies have shown that maximum leaf mass and area are scales as the −1.0 power of leafing intensity (LIV and LIM) across three different forest types (Sun et al., 2019a). Following the same logic as above, it is reasonable to suppose that the minimum leaf mass in twig has a less impact on total leaf mass. Assuming that twigs can sustain a constant total leaf mass, the leafing intensity changes in twig may be governed by the trade‐off between *M*_min_ and *M*_max_. For example, it follows *M*_max_ (LIV or LIM)^−1.0^, assuming that *M*_min_ ∞ *M*_max_^α^, and can be recast as *M*_min_ ∞ (LIV or LIM)^−α^. Thus, the exponents of leaf mass changes (from *M*_min_ to *M*_max_) versus leafing intensity (LIV or LIM) should follow the ranges between −α and −1.0, and thus constrained to be ≤−1.0. Based on these studies, we hypothesized (a) that the broad scaling scope of leaf size versus leafing intensity is tightly correlated with the biomass allocation in *M*_min_ and *M*_max_, and (b) the numerical value of the broad scaling exponent in deciduous and evergreen both are constrained to be ≤−1.0.

In order to test these hypotheses, we used the maximum and minimum leaf dry mass and leafing intensity (leaf number per stem volume or stem mass) of twigs from 123 species (66 evergreen species and 57 deciduous species) co‐occurring in evergreen and deciduous subtropical forests.

## 2 MATERIALS AND METHODS

### 2.1 Study site

Data were collected between 2016 and 2017 from two sites, Yangjifeng National Nature Reserve (YJF, 117°11′30″–117°28′40″E, 27°51′10″–28°02′20″N) and Wuyishan National Nature Reserve (WYS, 117°39′30″–117°55′47″E, 27°48′11″–28°00′35″N) of Jiangxi Province, southeastern China. The former had one 25‐ha plot, whereas the latter had three plots (0.12 ha each). The mean annual temperature ranged from 11.2 to 16.8°C; the mean annual precipitation was 1,900–2,200 mm (Table 1). In both sites, the soil type is primarily a subtropical mountain soil in both sites and derived from acidic crystalline rock weathering mainly composed of granite, granite porphyry, and gneiss. The dominant species in the Yangjifeng National Nature Reserve plots were *Castanopsis fargesii*, *Alniphyllum fortunei*, *Litsea cubeba*, *Castanopsis carlesii*, *Elaeocarpus sylvestris*, and *Schima superba*. The plots had a tree density of 703.52 trees ha^−1^, with the mean tree height (DBH > 5 cm) of 8.54 m, and mean trunk diameter at breast height (DBH) was 11.77 cm. The dominant species (e.g., *Rhododendron simiarum*, *Schima superba*, *Cyclobalanopsis glauca*, *Symplocos sumuntia*, *Cyclobalanopsis multinervis*, *Tsuga chinensis*, *Taxus wallichiana*, *Acer elegantulum*, *Illicium angustisepalum*) and site characteristics of the Wuyishan National Nature Reserve were listed in Sun et al. (2019a,b).

**TABLE 1:** Climatic conditions for two sampling sites YJF and WYS in mountain forests. “No. of species” indicates the total number of species in each plot, including 20 overlapping species

Site	Plot total area (ha)	Forest type	Elevations (m)	MAP (mm)	MAT (°C)	No. of species
YJF	25	EF	332	1900	16.8	68
WYS	0.12	EF	1,319	2000	14.5	32
	0.12	MF	1697	2,200	12.3	20
	0.12	DF	1818	2,200	11.2	23

### 2.2 Twig sampling

A total of 68 and 75 species were sampled from the two sites (Table 1). The total number of sampled species was 123 (including 20 overlapping species) belonging to 92 genera of 53 families. In August 2016 and 2017, three to five current‐year undamaged, healthy twigs from three individuals of each species were randomly selected. All of the leaves (with petioles) on each twig were removed and counted (*N*_L_). Twig diameter (*D*) and length (*L*) were measured using a vernier caliper, with an accuracy of 0.1 mm (Milla, 2009). The stem volume of each twig (*V*_stem_, mm^3^) was estimated using *L* and *D* assuming stems are cylindrical. Stems and leaves were brought to the laboratory where they were oven‐dried at 75°C to determine total leaf mass (*M*_leaf_) and stem mass (*M*_stem_). Each leaf of each twig was subsequently scanned, and its area was calculated using the ImageJ software (ImageJ 1.2v; National Institutes of Health, USA). Then, we multiplied the area of the largest and smallest leaf per twig by leaf mass per area (LMA, total leaf mass divided by total leaf area) to estimate the maximum and minimum leaf mass per twig, respectively. The volume‐based leafing intensity (LIV) is here defined as *N*_L_/*V*_stem_; the mass‐based leafing intensity (LIM) is here defined as *N*_L_/*M*_stem_.

### 2.3 Data analysis

All the data were log‐transformed to fit a normal distribution before statistical analysis. Regression analyses showed that log–log‐linear correlations between the variables of primary interest conformed to the equation log(*y*_1_) = log(*β*) + *α*log(*y*_2_), where *β* is the normalization constant, *α* is the scaling exponent, and *y*_1_ and *y*_2_ are interdependent variables of interest. Model Type II regression was used to determine the numerical values of *β* and *α* using the (Standardised) Major Axis Estimation package “smatr” version 3.4‐3 (Warton et al., 2012) in R‐4.0 software. The data from species showing no statistically significant differences in the numerical values of β and α were pooled to determine a common scaling exponent using the standardized major axis package (Warton et al., 2006, 2012) in R‐4.0. The significance level for testing slope heterogeneity was *p* < .05 (i.e., slope heterogeneity was rejected when *p* > .05).

## 3 RESULTS

### 3.1 The leaf size variation in twigs

The minimum leaf mass variation in twigs spanned four orders of magnitude (i.e., 0.0003 g for *Taxus chinensis* and 1.6087 g for *Castanopsis tibetana*). The maximum leaf mass spanned three orders of magnitude (i.e., 0.0032 g for *Taxus chinensis* and 1.8889 g for *Castanopsis tibetana*) across the 123 woody species (see File S1).

### 3.2 The scaling exponents among the *M*_IL_, *M*_min_, and *M*_max_

**FIGURE 1:**
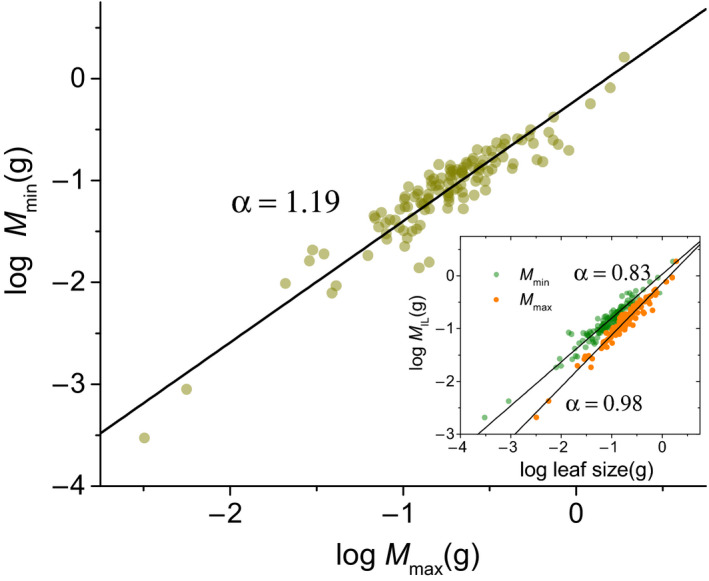
Bivariate plots among the minimum (*M*_min_) and maximum (*M*_max_) individual leaf mass and the mean individual leaf mass (*M*_IL_) variations in twig

Across entire data, the *M*_min_ scaled allometrically with respect to *M*_max_, with a slope of 1.19 (95% CI = 1.11–1.27, *r* ^2^ = .87, *P*_1.0_ = .001), which is significantly larger than 1.0 (Figure 1). However, the scaling exponents shifted from significantly lower than 1.0 for the relationships between *M*_IL_ and *M*_min_ (i.e., 0.83, 95% CI = 0.79–0.87, *r* ^2^ = .93, *P*_1.0_ = .001) to 1.0 for the relationships between *M*_IL_ and *M*_max_ (i.e., 0.98, 95% CI = 0.94－1.02, *r* ^2^ = .95, *P*_1.0_ = .31; Figure 1).

### 3.3 The shifting of scaling exponents between leaf size‐number

**FIGURE 2:**
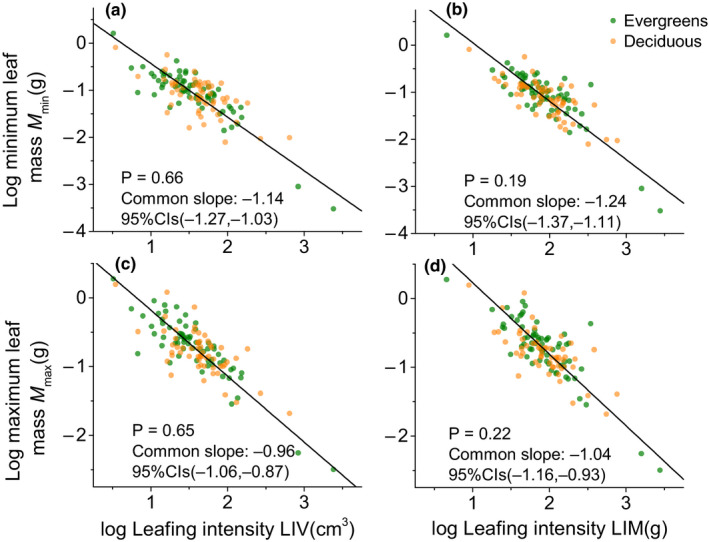
Bivariate plots of leafing intensity versus leaf size variations in twig. (a) the minimum leaf mass versus leafing intensity based on stem volume; (b) the minimum leaf mass versus leafing intensity based on stem mass; (c) the maximum leaf mass versus leafing intensity based on stem volume; (d) the maximum leaf mass versus leafing intensity based on stem mass

Overall, *M*_min_ scaled as −1.13 (95% CI = −1.26 to −1.02, *r* ^2^ = .64) and −1.24 (95% CI = −1.38 to −1.12, *r* ^2^ = .67) with LIV and LIM, respectively (Table 2), and were statistically different from −1.0 (*P*_−1.0_ = .02, .001, respectively) but were not different from −1.19 (the −*α* of *M*_min_ versus *M*_max_, *P*_−1.19_ = .38 and .42, respectively). Meanwhile, across entire dataset, *M*_max_ scaled as −1.05 (95% CI = −1.17 to −0.94, *r* ^2^ = .67) and −0.96 (95% CI = −1.06 to −0.86, *r* ^2^ = .63) with leafing intensity (LIV and LIM, respectively) (Table 2) and were not statistically different from −1.0 (*P*_−1.0_ = 0.38 and 0.41, respectively). More importantly, for different leaf habit groups, the common scaling exponents were found which shifted significantly from lower than −1.0 for *M*_min_ versus leafing intensity (i.e., −1.14 and −1.24, respectively) to −1.0 for the relationships between *M*_max_ versus leafing intensity (i.e., −0.96 and −1.04, respectively; Figure 2).

**TABLE 2:** Summary of regression slopes and *Y*‐intercepts (α and log β, respectively) for minimum (*M*_min_) and maximum individual leaf mass (*M*_max_) versus leafing intensity (calculated on the basis of stem volume LIV, and mass LIM, respectively) for data collected from twigs of 123 species

log y_1_ versus log y_2_	Forests	*n*	*α* (95% CI)	log β (95% CI)	*r* ^2^	*P_−_*_1.0_
*M*_min_ versus LIV	Evergreen	66	−1.16 (−1.31, −1.02)	0.73 (0.50, 0.96)	0.75	0.02
	Deciduous	57	−1.10 (−1.34, −0.90)	0.67 (0.29, 1.04)	0.43	0.36
	All	123	−1.13 (−1.26, −1.02)	0.71 (0.50, 0.91)	0.64	0.02
*M*_min_ versus LIM	Evergreen	66	−1.30 (−1.48, −1.14)	1.43 (1.09, 1.77)	0.72	0.001
	Deciduous	57	−1.13 (−1.34, −0.95)	1.06 (0.67, 1.44)	0.59	0.16
	All	123	−1.24 (−1.38, −1.12)	1.29 (1.04, 1.55)	0.67	0.001
*M*_max_ versus LIV	Evergreen	66	−0.97 (−1.09, −0.86)	0.80 (0.61, 0.99)	0.78	0.64
	Deciduous	57	−0.92 (−1.12, −0.76)	0.72 (0.42, 1.02)	0.49	0.41
	All	123	−0.96 (−1.06, −0.86)	0.77 (0.61, 0.94)	0.67	0.38
*M*_max_ versus LIM	Evergreen	66	−1.10 (−1.25, −0.96)	1.39 (1.10, 1.68)	0.71	0.18
	Deciduous	57	−0.95 (−1.14, −0.79)	1.05 (1.70, 1.40)	0.52	0.59
	All	123	−1.05 (−1.17, −0.94)	1.27 (1.04, 1.49)	0.63	0.41

## 4 DISCUSSION

### 4.1 The broad scope of scaling exponents between leaf size–number

Consistent with the hypothesis, the *M*_min_ scaled allometrically with 1.19 power to *M*_max_ in twigs across the 123 species. The numerical value of the scaling exponent of minimum leaf mass and leafing intensity was not statistically different from the −*α* of *M*_min_ versus *M*_max_, so that the lower limit to scaling of leaf mass and leafing intensity in twig has been established. Given that *M*_max_ scaled with leafing intensity was not statistically different from −1.0, therefore, our results validate the broad scope (constrained to be ≤−1.0) tightly correlated with the biomass allocation between the minimum leaf and maximum leaf in twig. Second, the phenomenon revealed by our data indicates constraints on the relationships among the total leaf number, the maximum individual leaf mass, and minimum individual leaf mass for subtropical mountain forest species. It is plausible that these constraints indicate a trade‐off between the carbon benefits and the costs for leaf size. For example, studies have shown that increases in leaf area generally do not result in proportional increases in leaf mass (Milla & Reich, 2007; Niklas & Cobb, 2008; Niklas et al., 2007; Sun et al., 2017). Similarly to the “diminishing returns” hypothesis (Niklas et al., 2007), our data reveal the scope of the lower and upper limits of leaf mass versus leafing intensity, which may indicate that gains in leaf number per unit stem size do not keep pace with increasing individual leaf mass, because the increase in leaf number may directly correspond to increased support investments (i.e., petioles) in twigs rather than lamina mass (Fan et al., 2017; Li et al., 2008; Niinemets et al., 2006). Constrained by the maximum leaf support investments, leafing intensity may decrease with increasing leaf mass (Milla, 2009; Xiang & Liu, 2009). Additionally, our results suggest that minimum leaf mass scaled as 1.19 power with respect to the maximum leaf mass (Figure 1), which indicate that plant preferentially allocate the resource to the minimum leaf at given a maximum leaf mass on twigs. Therefore, the minimum leaf mass investment in twig would significantly affect leaf number per stem size. Thus, the broad scope of scaling exponents between leaf size–number could be determined by the boundary through the biomass allocation between the *M*_min_ and *M*_max_.

In particular, our analyses used the minimum individual leaf mass and maximum individual leaf mass per twig, which was calculated without using leaf number in any way to avoid prior works' statistical bias, that is, total leaf mass divided by leaf number versus leaf number per stem size. Then, this approach directly establishes the boundary limit of leaf mass that supports the ability of a stem to provide resources. To the best of our knowledge, the data presented here are the first to quantify the general scope of leaf size–number and first to be embedded within and driven by a simple biomass allocation model. The exponents ranging from 0.83 to 0.98 between the mean individual leaf mass versus *M*_min_ and *M*_max_ also suggest that the mean individual leaf mass does not keep pace with increasing minimum leaf mass in current‐year twigs, but proportionally increases with the maximum leaf mass. As we mentioned at the beginning of this text, although the maximum leaf mass has a significant contribution to total leaf mass, the minimum leaf mass may provide a more flexible expansion space for the number of leaves within and across the different species. Therefore, the trade‐off between leaf size and leafing intensity is deeply influenced by the minimum leaf mass versus maximum leaf mass allocation in twigs.

### 4.2 Is the broad scope change with leaf habit?

When the leaf habit groups of the dataset were considered in isolation, the exponents of *M*_min_ scaled with respect to leafing intensity were significantly <−1.0 in evergreen species and ≈−1.0 in deciduous (Table 2), which is consistent with the exponent of mean leaf mass versus leafing intensity reported by Milla (2009). But, *M*_max_ scaled isometrically with leafing intensity across the evergreen and deciduous species (Table 2). In other words, the deciduous species, not evergreens, gains in leaf number per unit stem size do not keep pace with increasing the minimum leaf mass. For any given maximum individual leaf mass, deciduous species produce some leaves which is not different from evergreens (Table 2, Figure 2c,d) in the subtropical forest communities. This phenomenon first indicates that deciduous species prefer to choose the quick investment–return strategies (Zhao, Ali, & Yan, 2017). For instance, deciduous species usually have a simple branch structure and invest more resources in branches' growth than leaves (Li et al., 2008). In contrast, smaller leaf size but high leafing intensity in evergreen species may indicate that support investment (i.e., petioles) and bud compensation mechanism is too costly (Dombroskie et al., 2016; Niinemets et al., 2006), which is essential for leaf regrowth and recovery of evergreen species when it may easily suffer leaf area loss in longer leaf life span (i.e., insect herbivory) (Coley & Barone, 1996). Thus, the deciduous species were grouped on the acquisitive side based on the lower leafing intensity. In comparison, the evergreen species through the higher leafing intensity as compensation mechanisms were grouped on the conservative side in this study.

Secondly, due to the maximum leaf mass expansion limited in the twig, the scaling scope of leaf size versus leaf number may be converged in the deciduous and evergreen species. We offer a plausible explanation because the maximum leaf mass's distribution characteristics showed similar degrees of leptokurtosis and asymmetry between evergreen and deciduous (see File S2). The results indicate that the potential maximum total leaf mass (maximum leaf mass × leaf number) might be usually constrained by stem hydraulic or mechanical traits (Fan et al., 2017; Westoby et al., 2002) in different leaf habit groups. For example, prior studies suggest that leaf size generally scaled positively with respect to the diameter of the stem (Sun et al., 2019b; White, 1983), and in some plants, both mechanical and hydraulic constraints may be responsible for the limited maximum leaf size (Niinemets et al., 2002). Thus, constrained by maximum leaf size expand, the upper limit for the range of leaf size and number may be insensitive to different leaf habit groups.

Indeed, although different leaf habit groups differ in their minimum leaf mass versus leafing intensity adjustment strategies, the scaling scope variation of leaf size and number in twigs did not differ between deciduous and evergreen species, because the *M*_min_ versus leafing intensity and *M*_max_ versus leafing intensity have a common scaling exponent between the 66 evergreens and 57 deciduous species, and both are significantly ≤−1.0 (Figure 2). However, for different functional groups, whether this study's hypothesis is valid or not needs more empirical exploration. This study only has two coniferous species (*Tsuga chinensis* and *Taxus chinensis*) and two compound‐leaf species (*Gleditsia japonica* and *Albizia kalkora*). Thus, future research should include plants from different groups to investigate and capture the relationships among the leaf‐twig resource boundary on a broader scale.

In conclusion, leaf size and leafing intensity are essential for understanding plant performance and plasticity strategies. Our results report the definitive scope is constrained to be ≤−1.0 within and across the deciduous and evergreen species. The biomass allocation between maximum leaf and minimum leaf in twig establishes a lower limit for the range of leafing intensity strategies. Meanwhile, limited by the maximum leaf mass expand, the broad scope range between leaf size and number may not be sensitive to leaf habit groups in the subtropical mountain forest.

## CONFLICT OF INTEREST

The authors declare that they have no conflict of interest.

## AUTHOR CONTRIBUTION

**Jun Sun:** Conceptualization (lead); Data curation (equal); Formal analysis (lead); Investigation (equal); Methodology (equal); Project administration (lead); Resources (equal); Software (equal); Supervision (equal); Validation (equal); Visualization (equal); Writing‐original draft (lead); Writing‐review & editing (lead). **Xiaoping Chen:** Data curation (equal); Formal analysis (equal); Funding acquisition (equal); Investigation (equal); Methodology (equal). **Mantang Wang:** Conceptualization (equal); Data curation (equal); Formal analysis (equal); Investigation (equal); Methodology (equal); Project administration (equal). **Jinlong Li:** Conceptualization (equal); Data curation (equal); Formal analysis (equal); Resources (equal); Software (equal); Validation (equal); Visualization (equal). **Quan‐ling Zhong:** Conceptualization (equal); Data curation (equal); Formal analysis (equal); Investigation (equal); Project administration (equal); Supervision (equal); Validation (equal); Visualization (equal). **Dongliang Cheng:** Conceptualization (lead); Data curation (equal); Formal analysis (equal); Funding acquisition (lead); Investigation (equal); Methodology (equal); Project administration (equal); Resources (equal); Software (equal); Supervision (lead); Validation (equal); Visualization (equal); Writing‐original draft (equal); Writing‐review & editing (equal).

### OPEN RESEARCH BADGES

This article has earned an Open Data Badge for making publicly available the digitally‐shareable data necessary to reproduce the reported results. The data is available at https://doi.org/10.5061/dryad.wpzgmsbk5.

## Supporting information

File S1‐S2

## Data Availability

Data from this manuscript were archived in the publicly accessible repository Dryad (https://doi.org/10.5061/dryad.wpzgmsbk5).
