# Supplementary material for: Application of leaf size and leafing intensity scaling across subtropical trees
Source: Ecol Evol. 2020 Nov 12;10(23):13395–402. doi: 10.1002/ece3.6943 (PMC7713914; doi:10.1002/ece3.6943)
Supplement: Supplementary file 1 — File S1‐S2 [file ECE3-10-13395-s001.doc]

**Supplementary information**

S1. Individual leaf mass traits for the 123 species in the twig level. The minimum (*M*_min_) and maximum (*M*_max_) individual leaf mass and the leafing intensity (LIV and LIM).

| Species | Leaf habit | *M*_min_ (g) | *M*_max_(g) | LIV ( n·mm^-3^) | LIM ( n·g^-1^) |
| --- | --- | --- | --- | --- | --- |
| *Castanea_mollissima* | Deciduous | 0.1444 | 0.2346 | 56.0858 | 26.6165 |
| *Itea_chinensis* | Evergreen | 0.2535 | 0.3880 | 28.6634 | 44.1022 |
| *Syzygium_buxifolium* | Evergreen | 0.0427 | 0.0689 | 150.0459 | 213.7185 |
| *Aralia_chinensis* | Deciduous | 0.5648 | 1.2057 | 16.2012 | 46.7737 |
| *Styrax_grandiflorus* | Deciduous | 0.1350 | 0.2233 | 40.8159 | 191.0611 |
| *Ilex_purpurea* | Evergreen | 0.1251 | 0.2190 | 22.3211 | 80.0366 |
| *Rhododendron_simsii* | Deciduous | 0.0208 | 0.0300 | 130.2938 | 201.4144 |
| *Quercus_serrata* | Deciduous | 0.1227 | 0.3097 | 42.1222 | 40.5592 |
| *Liquidambar_formosana* | Deciduous | 0.1699 | 0.3761 | 37.5467 | 63.8946 |
| *Eurya_muricata* | Evergreen | 0.0756 | 0.1812 | 66.9902 | 110.1658 |
| *Castanopsis_tibetana* | Evergreen | 1.6087 | 1.8889 | 3.2422 | 4.5316 |
| *Betula_luminifera* | Deciduous | 0.1407 | 0.1876 | 82.9284 | 43.4841 |
| *Illicium_henryi* | Evergreen | 0.1548 | 0.2503 | 27.4071 | 91.2846 |
| *Machilus_thunbergii* | Evergreen | 0.1967 | 0.9041 | 11.0073 | 44.7790 |
| *Sloanea_sinensis* | Evergreen | 0.0676 | 0.2235 | 39.0288 | 177.2387 |
| *Ternstroemia_gymnanthera* | Evergreen | 0.1525 | 0.6388 | 15.6127 | 45.1212 |
| *Ilex_elmerrilliana* | Evergreen | 0.0907 | 0.2152 | 30.9385 | 167.2171 |
| *Machilus_leptophylla* | Evergreen | 0.2554 | 0.4578 | 23.7173 | 68.4214 |
| *Platycarya_strobilacea* | Deciduous | 0.0226 | 0.1019 | 81.5049 | 153.7909 |
| *Adinandra_millettii* | Deciduous | 0.2735 | 0.5354 | 15.4127 | 59.8204 |
| *Dalbergia_hupeana* | Deciduous | 0.0520 | 0.0958 | 64.7244 | 143.9928 |
| *Loropetalum_chinense* | Evergreen | 0.0450 | 0.0680 | 105.5059 | 174.7446 |
| *Populus_canadensis* | Deciduous | 0.1409 | 0.2859 | 50.6946 | 142.5536 |
| *Lindera_chienii* | Deciduous | 0.0991 | 0.1454 | 45.2080 | 77.6966 |
| *Daphniphyllum_macropodrum* | Evergreen | 0.0526 | 0.2228 | 42.2080 | 65.0699 |
| *Castanopsis_fargesii* | Evergreen | 0.1053 | 0.1669 | 49.0812 | 80.9916 |
| *Melia_azedarach* | Deciduous | 0.0590 | 0.1321 | 129.3107 | 214.5755 |
| *Castanopsis_sclerophylla* | Evergreen | 0.2058 | 0.3702 | 17.6672 | 25.7284 |
| *Nyssa_sinensis* | Deciduous | 0.0905 | 0.1615 | 47.6194 | 37.1346 |
| *Symplocos_stellaris* | Evergreen | 0.3148 | 0.5443 | 7.8758 | 31.9492 |
| *Chimonanthus_nitens* | Evergreen | 0.0600 | 0.1264 | 61.9625 | 74.9571 |
| *Diospyros_morrisiana* | Deciduous | 0.0618 | 0.1834 | 41.2541 | 150.0111 |
| *Rhododendron_ovatum* | Evergreen | 0.0139 | 0.1236 | 89.7659 | 181.0342 |
| *Armeniaca_mume* | Evergreen | 0.0265 | 0.0805 | 153.4004 | 120.9854 |
| *Castanopsis_carlesii* | Evergreen | 0.1039 | 0.1501 | 76.3750 | 92.9881 |
| *Phoebe_bournei* | Evergreen | 0.0526 | 0.1419 | 23.2596 | 50.7827 |
| *Schima_superba* | Evergreen | 0.0982 | 0.2972 | 25.1316 | 65.8529 |
| *Choerospondias_axillaris* | Evergreen | 0.0751 | 0.1826 | 55.5310 | 77.6106 |
| *Alniphyllum_fortunei* | Evergreen | 0.4183 | 0.7385 | 21.6484 | 28.0149 |
| *Celtis_sinensis* | Deciduous | 0.0583 | 0.1289 | 65.7911 | 149.1452 |
| *Randia_cochinchinensis* | Evergreen | 0.0817 | 0.1987 | 66.4645 | 129.4644 |
| *Elaeocarpus_japonicus* | Evergreen | 0.1312 | 0.4259 | 14.2859 | 25.0431 |
| *Machilus_velutina* | Evergreen | 0.2249 | 0.7874 | 15.1445 | 48.2207 |
| *Manglietia_yuyuanensis* | Evergreen | 0.1070 | 0.2700 | 30.5067 | 96.2083 |
| *Litsea_cubeba* | Deciduous | 0.0301 | 0.0797 | 74.5586 | 98.8398 |
| *Elaeocarpus_sylvestris* | Evergreen | 0.0984 | 0.2624 | 27.2690 | 78.9173 |
| *Gleditsia_japonica* | Deciduous | 0.0098 | 0.0209 | 645.0233 | 549.7471 |
| *Chimonanthus_praecox* | Evergreen | 0.0407 | 0.0897 | 110.7066 | 106.5193 |
| *Engelhardtia_fenzelii* | Evergreen | 0.2333 | 0.3472 | 38.1469 | 64.1042 |
| *Lithocarpus_glaber* | Evergreen | 0.2306 | 0.5646 | 28.3141 | 41.5333 |
| *Dendropanax_dentiger* | Evergreen | 0.1599 | 0.5930 | 12.6365 | 43.5556 |
| *Fagus_longipetiolata* | Deciduous | 0.1285 | 0.3263 | 36.5184 | 51.2985 |
| *Castanopsis_eyrei* | Evergreen | 0.1452 | 0.4327 | 38.5630 | 343.5012 |
| *Cornus_elliptica* | Evergreen | 0.0345 | 0.0948 | 119.4957 | 261.7676 |
| *Altingia_gracilipes* | Evergreen | 0.0691 | 0.1024 | 55.3398 | 153.7736 |
| *Rhus_chinensis* | Evergreen | 0.0802 | 0.2986 | 11.1145 | 61.9678 |
| *Myrica_rubra* | Evergreen | 0.0317 | 0.1125 | 79.9548 | 97.7712 |
| *Michelia_skinneriana* | Deciduous | 0.1114 | 0.1874 | 26.2450 | 103.0269 |
| *Cinnamomum_jensenianum* | Deciduous | 0.1213 | 0.1894 | 62.2276 | 86.9395 |
| *Toxicodendron_succedaneum* | Deciduous | 0.0552 | 0.1063 | 92.1382 | 72.7782 |
| *Diospyros_kaki* | Deciduous | 0.2485 | 0.7350 | 37.1961 | 24.3455 |
| *Mallotus_japonicus* | Deciduous | 0.8087 | 1.5675 | 3.4708 | 8.8997 |
| *Euscaphis_japonica* | Deciduous | 0.0733 | 0.1397 | 41.5102 | 131.2757 |
| *Populus_canadensis* | Deciduous | 0.1682 | 0.3291 | 14.8876 | 21.5430 |
| *Cerasus_discoidea* | Deciduous | 0.0692 | 0.1161 | 98.6496 | 100.4197 |
| *Camellia_oleifera* | Evergreen | 0.1944 | 0.3022 | 24.0159 | 156.8171 |
| *Cinnamomum_camphora* | Evergreen | 0.1194 | 0.2196 | 56.8532 | 134.4562 |
| *Phoebe_faberi* | Evergreen | 0.0733 | 0.1212 | 56.3842 | 157.9523 |
| *Castanea_henryi* | Deciduous | 0.1505 | 0.3424 | 21.2284 | 41.6597 |
| *Lithocarpus_iteaphyllus* | Evergreen | 0.1258 | 0.2732 | 32.8041 | 70.8060 |
| *Pterostyrax_corymbosus* | Deciduous | 0.1496 | 0.2312 | 43.2641 | 67.2781 |
| *Cerasus_pseudocerasus* | Deciduous | 0.0839 | 0.2049 | 59.6825 | 73.8257 |
| *Cyclobalanopsis_glauca* | Evergreen | 0.0938 | 0.1827 | 26.5286 | 59.1351 |
| *Ilex_wilsonii* | Evergreen | 0.0349 | 0.0722 | 87.0537 | 165.0160 |
| *Symplocos_sumuntia* | Evergreen | 0.1564 | 0.2881 | 26.2118 | 61.3699 |
| *Corylopsis_sinensis* | Deciduous | 0.0669 | 0.1397 | 45.9614 | 123.3245 |
| *Carpinus_viminea* | Deciduous | 0.0384 | 0.0789 | 64.8656 | 130.5385 |
| *Cornus_controversa* | Deciduous | 0.1321 | 0.2566 | 21.0653 | 44.4995 |
| *Cyclobalanopsis_gracilis* | Evergreen | 0.0706 | 0.1814 | 43.8252 | 82.3168 |
| *Ilex_ficoidea* | Evergreen | 0.0717 | 0.2575 | 31.9448 | 92.9698 |
| *Rhododendron_latoucheae* | Evergreen | 0.1284 | 0.1858 | 35.2380 | 56.0967 |
| *Erythroxylum_sinense* | Deciduous | 0.0184 | 0.0626 | 49.2704 | 136.4982 |
| *Itea_oblonga* | Evergreen | 0.1137 | 0.2005 | 13.9990 | 43.2910 |
| *Cyclobalanopsis_multinervis* | Evergreen | 0.1100 | 0.2261 | 27.0270 | 58.9716 |
| *Albizia_kalkora* | Deciduous | 0.0532 | 0.1802 | 183.4892 | 382.3670 |
| *Illicium_angustisepalum* | Evergreen | 0.2000 | 0.2676 | 14.6825 | 47.5277 |
| *Castanopsis_fargesii* | Evergreen | 0.1327 | 0.2742 | 25.3823 | 51.9459 |
| *Ilex_pedunculosa* | Evergreen | 0.0412 | 0.0838 | 46.6729 | 124.2655 |
| *Litsea_rotundifolia* | Evergreen | 0.1639 | 0.2532 | 22.0193 | 72.0461 |
| *Cinnamomum_pauciflorum* | Evergreen | 0.2270 | 0.3838 | 10.2566 | 24.4821 |
| *Symplocos_wikstroemiifolia* | Evergreen | 0.0428 | 0.1135 | 17.7139 | 59.6388 |
| *Cyclobalanopsis_myrsinifolia* | Evergreen | 0.1185 | 0.2182 | 20.9382 | 46.2896 |
| *Rhododendron_simiarum* | Evergreen | 0.2503 | 0.4841 | 14.1401 | 37.8956 |
| *Tsuga_chinensis* | Evergreen | 0.0003 | 0.0032 | 2414.3522 | 2757.8693 |
| *Litsea_pungens* | Deciduous | 0.0891 | 0.1456 | 19.0535 | 42.4764 |
| *Eurya_saxicola* | Evergreen | 0.0163 | 0.0287 | 113.3043 | 300.3412 |
| *Acer_palmatum* | Deciduous | 0.1133 | 0.1413 | 56.5850 | 77.5307 |
| *Viburnum_setigerum* | Deciduous | 0.1432 | 0.1604 | 18.8009 | 44.5964 |
| *Eurya_brevistyla* | Evergreen | 0.0792 | 0.1219 | 39.5798 | 92.2415 |
| *Taxus_chinensis* | Evergreen | 0.0009 | 0.0056 | 837.1405 | 1574.8885 |
| *Halesia_macgregorii* | Deciduous | 0.0551 | 0.1023 | 49.9917 | 109.1986 |
| *Camellia_fraterna* | Evergreen | 0.0190 | 0.0349 | 135.5858 | 248.7169 |
| *Cornus_hongkongensis* | Evergreen | 0.0869 | 0.1073 | 63.3492 | 141.3583 |
| *Sorbus_alnifolia* | Deciduous | 0.0655 | 0.1015 | 87.3135 | 202.7241 |
| *Rhododendron_fortunei* | Evergreen | 0.2962 | 0.6925 | 5.5863 | 17.9207 |
| *Acer_elegantulum* | Deciduous | 0.1246 | 0.1936 | 44.7591 | 72.1918 |
| *Meliosma_cuneifolia* | Deciduous | 0.0800 | 0.1598 | 24.2965 | 79.5066 |
| *Agapetes_lacei* | Deciduous | 0.0093 | 0.0409 | 269.0325 | 758.5169 |
| *Alpinia_japonica* | Deciduous | 0.0321 | 0.1005 | 41.9635 | 111.7239 |
| *Symplocos_paniculata* | Deciduous | 0.0513 | 0.1332 | 53.4962 | 120.6775 |
| *Symplocos_urceolaris* | Evergreen | 0.0885 | 0.1539 | 6.7646 | 30.9947 |
| *Clethra_barbinervis* | Deciduous | 0.0158 | 0.1414 | 31.7483 | 159.4138 |
| *Prunus_padus* | Deciduous | 0.0673 | 0.1840 | 33.9189 | 78.2015 |
| *Viburnum_sympodiale* | Deciduous | 0.1961 | 0.3233 | 6.8051 | 18.9329 |
| *Lindera_erythrocarpa* | Deciduous | 0.0272 | 0.1054 | 45.1274 | 130.9766 |
| *Ilex_tsoii* | Deciduous | 0.0484 | 0.0748 | 18.4317 | 39.4623 |
| *Lindera_obtusiloba* | Deciduous | 0.0364 | 0.1252 | 42.5433 | 127.2108 |
| *Magnolia_amoena* | Deciduous | 0.1013 | 0.1909 | 22.2524 | 89.9336 |
| *Photinia_beauverdiana* | Deciduous | 0.0765 | 0.2196 | 41.1051 | 61.7926 |
| *Acer_nikoense* | Deciduous | 0.0334 | 0.1194 | 16.4068 | 65.8108 |
| *Zanthoxylum_simulans* | Deciduous | 0.0079 | 0.0388 | 93.5558 | 318.4148 |
| *Fraxinus_chinensis* | Deciduous | 0.1279 | 0.2057 | 19.2930 | 50.3774 |
| *Stewartia_gemmata* | Deciduous | 0.0649 | 0.1392 | 44.0533 | 106.7379 |

S2 Frequency distribution of species mean values of minimum leaf mass and maximum leaf mass for the whole compilation data set (a and b indicate evergreen species), (c and d indicate deciduous species). Indexes of asymmetry and kurtosis, along with their standard errors, are shown in the insets.
